# A Treatise on Pulmonary Consumption; Comprehending an Inquiry into the Causes, Nature, Prevention, and Treatment of Tuberculous and Scrofulous Diseases in General

**Published:** 1836-01

**Authors:** 


### Art. III.

*A Treatise on Pulmonary Consumption; comprehending an Inquiry into the Causes, Nature, Prevention, and Treatment of Tuberculous and Scrofulous Diseases in general.* B James Clark, m.d. f.r.s., &c. —8vo. *London*, 1835. Pp. 398.

The substance of the present treatise, it may be proper to mention, first appeared, about a year ago, in the Cyclopædia of Practical Medicine, in the form of an article entitled "Tubercular Phthisis." The favorable reception which it instantly met with induced Dr Clark to revise and enlarge it for separate publication. Much of it having been rewritten, and the whole of it improved, we can say with truth that, as it now stands, we are not acquainted with any single volume on the subject of tuberculous affections, which we should be so earnest in recommending to the diligent study of the profession, especially the younger members. In individual parts it may be surpassed by the admirable works of Laennec, Bayle, Louis, and Andral; but, as a text-book and guide to the inexperienced practitioner, we know none equal to it in general soundness and practical utility.

In common with all late writers, Dr. Clark restricts the term pulmonary phthisis to that form of consumption which depends on the existence and breaking down of tubercles in the lungs; and consequently the grand aim which he has in view is to obtain a correct knowledge of the origin, nature, and treatment of tubercular disease. How far he has been successful in attaining his aim, we shall proceed to enquire.

Two opinions are entertained in regard to the origin of tubercles. By some they are considered to be merely a local affection, and the result of local morbid action, with which the system sympathises only after the local disease has made considerable progress. By others they are held to be never produced by local irritation alone, *but to be invariably the consequence of a pre-existing unhealthy state of the constitution, without which the accidental causes which call them into being would have been entirely incapable of producing them.* Among the advocates of the latter opinion, Dr. Clark is perhaps the most distinguished.

If pulmonary tubercles be a purely local affection, depending entirely on the colds and trifling accidents to which they are commonly ascribed, it will be sufficient for the prevention that we guard against exposure to these causes, whereas, if they be really a secondary ailment, originating in and dependent upon a previously disordered state of the general health, no protection which is not equally powerful in preserving the health of the system at large will be adequate to their prevention.

That pulmonary tubercles are not the primary disease, but really the local results of a pre-existing constitutional disturbance, seems to be established by their whole history. Whether we consider them in their origin, their progress, their complications, their ultimate terminations, or the powerlessness of local treatment for their removal, we arrive directly and invariably at the same conclusion. Imperfect though our knowledge is, we are too well acquainted with the intractable nature of actual disorganization to allow us to hope for the speedy discovery of any remedy capable of removing tubercles, when they have once been formed; and therefore, if medical aid is to be of any avail, it must be in the detection and appropriate treatment of the disease in its first or constitutional stage, and not after delaying till actual disorganization forces the patient to apply for relief.

Such being the fundamental principle earnestly advocated by Dr. Clark, of the truth and importance of which we entertain a profound conviction, we need scarcely add, that we rejoice in the opportunity now offered to us of warmly recommending the present work, as containing the best exposition we have yet seen of "this the most important, but hitherto the most neglected, part of the subject;" and of expressing our entire concurrence with the author in believing it impossible, on any other principle, to arrive at "a sounder pathology of tuberculous diseases, or to establish a more rational and effectual mode of prevention, and a more successful method of treatment, than have hitherto prevailed." (P. 6.)

In investigating the *causes* by which phthisis is induced, every step of the progress will be found to afford additional evidence of its constitutional origin; and every circumstance to which its occurrence is usually ascribed will be discovered to be powerful, in almost exact proportion to the influence which it possesses in previously deteriorating the general health. So long as the constitution remains unimpaired, the ordinary exciting causes may come into active play again and again, during a whole lifetime, without producing consumption; but, the moment the tone of the system is seriously lowered by sedentary habits, insufficiency of food, impaired digestion, depression of mind, excessive study, or living in an unwholesome atmosphere, the liability becomes so much increased, that a very slight external cause will then suffice to induce the deposition of tuberculous matter. It is, as Dr. Clark acutely remarks, from overlooking this obvious distinction between the local and constitutional conditions, that many things are set down as active exciting causes of phthisis which have, in reality, little direct influence in its production; while others, of much more importance, are disregarded, merely because their immediate effect is on the general system, and not exclusively on the lungs. Catarrh, for example, is set down as a very frequent cause of consumption, and yet we know that it may occur, in every degree of frequency and severity, in an otherwise healthy subject, without inducing a single tubercle; and that, in point of fact, thousands suffer almost yearly from catarrh, who nevertheless live on to a good old age. In like manner inflammation is stated to be a cause, and yet we see every day numerous instances of perfect recovery, without even a threatening of phthisis supervening; and a similar remark is applicable to wet feet, exposure to cold, and all other accidental occurrences, to which, solely because they preceded its observable development, consumption is often ascribed.

It is perfectly true that a cold or an inflammation sometimes excites pulmonary consumption, and that its influence cannot be safely neglected. But then, as Dr. Clark is most earnest in inculcating, it has this effect only where the constitution is either naturally or accidentally predisposed to it; and but for this predisposed state, to which Dr. Clark gives the distinctive name of *tuberculous* or *strumous cachexia*, no such serious results would have followed. The predisposing cachexia then, rather than the catarrh, affords the true key to the production of the subsequent local effect; and ought first to engage our attention. That this is the proper view of the origin of phthisis, will become still more evident as we proceed. We shall find the various circumstances from which the cachexia derives its origin to be precisely those which are also most powerful in inducing the ultimate result—phthisis pulmonalis; thus clearly demonstrating that the predisposing condition is, in fact as well as a theory, the most influential agent in the production of the disease.

Dr. Clark mentions that tuberculous or strumous cachexia may exist from birth, or be acquired at almost any period of life from infancy to old age. When it exists at birth, it is generally a result of the unhealthy condition of one or both parents. Dr. Clark "regards it as one of the best established points in the etiology of the disease, that a parent labouring under tuberculous cachexia entails on his offspring a disposition to the same affection, proportioned, in general, to the degree of the disease under which he is labouring." This latter remark is so perfectly true, that we often observe the malady varying in intensity in the different members of the same family, according to its greater or less activity in the parents at the time of conception. Children born to parents who either have not attained or have passed the vigour of maturity, generally inherit the predisposition more strongly than those born under more favorable circumstances. We know a curious example of this fact in a family where, out of seventeen children, the first eight presented the appearance of scrofula: whereas of the last nine born, necessarily after the strength of the parents was somewhat shaken, not fewer than six were deeply marked with scrofulous sores, and a seventh died of dropsical effusion from glandular obstruction. Of the other two, one, the youngest of all, died in infancy, and the other of fever at the age of twenty.

The common notion is, that it is only parents actually suffering under scrofulous cachexia who can impart it to their offspring: but Dr. Clark takes great pains to shew that this is a mistake, and that any cause whatever which seriously deteriorates the general health may give rise to the tubercular constitution in such of the children as are produced during the continuance of the parental bad health. If, for example, a soundly constituted parent becomes subject to severe dyspepsia, gout, cutaneous disease, or depression of mind, or is obliged to live constantly in a confined and unhealthy atmosphere, the children born to him during that time will generally exhibit all the features of the scrofulous diathesis, as strongly as if the parent himself laboured under its influence. In some instances, the more favorable situation of the mother may modify the result, but the tendency will still be the same. In like manner children procreated during convalescence from fever, or other illness, generally shew traces of their unfavorable origin.

Of all the causes, however, which act primarily on the parents, Dr. Clark remarks, that "dyspepsia is the most fertile source of cachexia in every form," although it "may also originate in derangement of the various secretory and excretory functions, particularly that condition of them in which the effete matter is imperfectly carried off." (P. 223.) Nor is this to be wondered at; for disordered digestion, by affording imperfect materials for the formation of new blood, necessarily impairs the nutrition of the whole system; and thereby renders every organ and function more than usually susceptible of disturbance.

Other conditions in the parent may induce the tubercular constitution in the offspring; but the above examples are sufficient to illustrate the principle and demonstrate the reality of the importance attached to it by our author. Before quitting the subject, however, we cannot resist quoting the following summary of the various degrees in which the tuberculous constitution may exist at birth:

"1. Although it is a rare occurrence, the child at birth may present tubercles in one or more organs.

"2. The next degree of hereditary disease is that in which the infant is afflicted with tuberculous cachexia,—a state which requires very slight exciting causes to determine the deposition of tuberculous matter in some organ. The children of consumptive parents are not unfrequently born in this state, and often die of tuberculous disease during the period of infancy.

"3. Again, the child presents all the characters of the tuberculous or scrofulous constitution, and, without care, gradually lapses into a state of tuberculous cachexia, and dies of tuberculous disease. The greater number of scrofulous and consumptive cases which we meet with in childhood and youth are referrible to this degree of hereditary predisposition.

"4. In another class of cases, the child merely shews a predisposition to those functional derangements which generate the tuberculous constitution; more especially to that form of dyspepsia—the *strumous*—to which I have already referred, as capable of generating the tuberculous cachexia, and consequently of giving rise to every form of tuberculous or strumous disease. The cases of predisposition to consumption which come under this class are, according to my observation, the offspring of parents who have laboured under dyspepsia, gout, cutaneous, and other diseases *not* of a tuberculous nature. *They constitute the most numerous and the most remediable of the degrees of hereditary disease,* and yet their nature is least understood." (P. 226.)

We agree with the author in considering the subject as deserving more serious attention from the profession than it has yet received; but we may remark, that we perceive little distinction between the second and third heads in the preceding division.

The principal causes which give rise to tuberculous cachexia, and consequently to consumption, in those not hereditarily predisposed, are, according to our author, "improper diet, impure air, deficient exercise, imperfect clothing, inattention to cleanliness, abuse of spirituous liquors, and affections of the mind;" and the earlier in life these causes exist, the less can the individual withstand their influence. Hence the vast importance of a rational system of diet and regimen for children from infancy upwards, and of a thorough reform of our prevailing modes of education.

*Improper diet* is well known to be a fruitful source of scrofulous disease, especially in very early life, when nutrition is going on actively. Its impropriety, however, may consist in *excess,* and in *quality,* as well as in *deficiency* of food. The lower classes suffer most from deficiency and inferior quality, and the higher from excess and too stimulating a quality. But among the poor there is usually added a combination of other causes, such as bad air, neglect of cleanliness, and want of clothing, which necessarily aggravate the want of proper food, and leave it difficult to determine the exact amount of its separate influence; but that this influence is great must be admitted by every one who has practised among the poor, particularly in seasons of scarcity. In the summer of 1809, when the children of the Dublin House of Industry were fed almost exclusively on vagetables and butter-milk, scrofula became extremely prevalent among them, and was ascribed by Mr. Carmichael partly to the faulty diet, and partly to impure air and deficient exercise. By substituting half a pound of animal food every alternate day, and a quart of boiled sweet milk daily, for the butter-milk and insufficient vegetable aliment, and improving the digestive functions by alteratives and tonics, "the amendment produced was obvious and rapid," and "there was not one instance of the disease afterwards making progress," although "the children unavoidably remained, while under treatment, exposed to the same impurity of air and want of exercise."* In these examples, the influence of diet is placed beyond a doubt.

But *want of fresh air and exercise* is even still more productive of tuberculous cachexia than deficiency of food; and accordingly, in workhouses, manufactories, and confined and crowded cities with narrow streets, where the air and the light of day scarcely ever enter, the diseases dependent on this state of the constitution are very prevalent. Baudelocque, indeed, goes so far as to say that impure air is the only cause of scrofula; and, when we consider that respiration is essential to the ultimate conversion of food into nutritive blood, we cannot wonder that the want of fresh air should have great influence in lowering the general tone, and generating the tubercular predisposition. On this point, also, Mr. Carmichael furnishes some decisive evidence. Out of thirty girls attending the Bethesda school, in Dublin, in 1809, *all fed and clothed in the best manner,* six were badly affected with scrofula, although not one of them presented any appearance of the disease on her admission. It appeared, on enquiry, that, from their being no play-ground attached to the institution, the children were obliged to remain in the school or bed-rooms during play-hours, without any opportunity of getting exercise in the open air; and that, in consequence, their health speedily began to suffer. The very same thing occurred in St. Thomas's parochial school, where *seven* out of twenty-four girls,—also well fed, clothed, and lodged,—were attacked by scrofula, apparently in consequence of having been kept "perpetually within doors, at their school-books," during the preceding winter and spring, owing to an inundation of their play-ground.

* See Carmichael's "Essay on the Nature of Scrofula," pp. 89, 90.

Not one of them had the disease when admitted, nor were any affected until they were deprived of air and exercise, although their diet was previously worse than when scrofula appeared. In the male and female asylum of the Dublin House of Industry, already alluded to, when speaking of diet, scrofula was formerly so prevalent as to be considered contagious by the attendants; but then it was so crowded by nearly five hundred children, that in one ward of moderate height, sixty feet long by eighteen broad, there were thirty-eight beds, each containing three children! Even during the day, most of the children were either confined in their sleeping apartment or crowded, to the number of several hundreds, in the same school-room. On opening the door of the female apartment in the morning, the matron found it impossible to endure the effluvia. Is it surprising that in such circumstances scrofula should have prevailed?

These facts (and we could mention others of a similar tendency, regarding our own charitable institutions,) are full of practical instruction, both as explaining the sources of the tubercular diathesis, and as suggesting means for its prevention and cure. Were it necessary, we might adduce additional evidence of the same kind from Lepelletier's prize essay;* but to that work it will be sufficient to refer.

A sedentary mode of life tends strongly to give rise to tuberculous cachexia, both from the absence of muscular exercise, and from the confinement to a close and impure air, which it necessarily involves. The whole animal system having been arranged by the Creator for a life of activity, the immediate effect of indolence and sedentary habits is to lower the activity of nutrition, and, as a necessary consequence, to impair the vigour of the digestive functions. Females, students, and literary persons, often induce an unsound state of constitution, from this cause, added, in the cases of the second and third, to too much mental excitement. But *excessive* bodily exertion is not less unfavorable to health, particularly before the age of maturity; and many youths do themselves irreparable injury in this way, during shooting or walking excursions, especially when growth is going on rapidly. Under such circumstances tubercular disease is often stirred up by causes which, under better management, would have had no such effect.

The *state of the mind*, also, is extremely influential in inducing the tuberculous constitution. If the mind be harassed by care, depressed by grief, or even exhausted by incessant exertion, the general health begins immediately to suffer: the stomach and bowels become disordered, nutrition languishes, a sallow paleness takes the place of the ordinary complexion, and disease is excited in the lungs, the mesentery, or any other organ, by the slightest local

* Traité complet de la Maladie Scrophuleuse, par Lepelletier de la Sarthe.—Paris.

irritation. Debility and a consciousness of impaired health are all that is felt for a time; but, as they do not incapacitate the individuals for business, they are too commonly neglected till they terminate in phthisical or other disease. It is to urge the early observance and requisite treatment of this really the first and only curable stage of tubercles, that the efforts of Dr. Clark are so strenuously and properly dedicated.

But, as remarked by our author,

"Of the various phenomena presented by a person strongly predisposed to or labouring under tuberculous disease, *a congestive state of the abdominal venous circulation* will, I believe, be found, on close examination, to be one of the most constant. In children originally of a strumous habit, we observe a constant disposition to this congested state of the abdominal circulation; and, unless we succeed in obviating it, they become tuberculous, and die early in life. In youth we find the same state of congestion as a precursor of tuberculous cachexia: at this age it manifests itself often by epistaxis, by hemoptysis, and even by hemorrhoids; but it is during the middle period of life, from thirty-five to fifty, that it is accompanied with more marked symptoms, such as dyspepsia and its various concomitants, which exist often for a very considerable time, and not unfrequently obscure the pulmonary affection till tuberculous disease has made extensive progress." (P. 263.)

Dr. Clark adds, that this congestive state was well known to the older writers, under the name of *abdominal infarctus;* and our experience leads us to think the weight attached to it by the author not over-rated. As a further corroboration, it may be mentioned that both Lepelletier and Carmichael notice the rise of scrofula from similar abdominal derangement. Lepelletier, indeed, contends that scrofula is an affection solely of the nutritive system; and in evidence of the fact, endeavours to shew that all its exciting causes may be arranged under three heads, according to the mode in which each affects the process of nutrition: viz. 1st, those which impair the assimilating action; 2dly, those which present insufficient elements of nutrition, such as bad diet and impure air; and, 3dly, those which impede healthy excretion.

The habitual use of *stimulating and exciting food and drink,* in too large quantity, is a frequent cause of the tubercular diathesis among the higher classes of society. When to this is added the depressing influence of late hours, close and heated rooms, vitiated air, mental inanity, insufficient exercise, and inadequate clothing, there is little difficulty in accounting for the appearance of the strumous constitution among the rich as well as among the poor; for the ultimate result differs little, whether the digestive functions suffer from deficiency or from excess of food.

The influence of *dress,* also, is very apparent. Among the poor, the want of adequate clothing aggravates every ailment. Among the peasantry of the northern and middle governments of Russia, the protecting power of sufficient clothing, and living much in the open air, is, according to Sir A. Crichton's account, not less striking. Scrofula, he says, is extremely prevalent in these districts; but, from the peasants being warmly clothed, and much out of doors, its ravages are confined almost entirely to the external glands; and, except in public schools, and among those who adopt the European dress and fashions, the lungs generally remain sound. In England, on the other hand, where a large proportion of the labouring classes spend their days in the confined air of our manufactories and workshops, and, when exposed to the weather, are very inadequately clothed, the pulmonary affection is far more commonly met with; and the horrible disfiguration of the surface, so frequent in Russia, is comparatively rare. In the same way, when it was the fashion, some years ago, for English women to dress very lightly, even in the depth of winter, consumption carried off numbers, who presented no external marks of scrofula, and who might have been saved by more rational conduct.

Under the head of *Statistics of Consumption*, Dr. Clark communicates much additional information on the origin of tuberculous cachexia, and which, in a practical point of view, might have been more usefully discussed when treating of the causes. The whole of it tends, like what has preceded, to demonstrate the grand principle of his work, that tubercles are not a local affection, but the results of constitutional disorder, and never make their appearance until the general health has been more or less deteriorated.

It appears, for example, from the best statistical tables, that tubercles rarely, if ever, occur at birth, except where either actual illness or a strong predisposition exists in the parents. After the end of the second year, however, when bad nursing, teething, improper diet, and confinement in an unfavorable locality, have had time to weaken the general system, and especially the functions of digestion and assimilation, they are very common; so much so, that, from the comparison of various observations, Dr. Clark thinks it may "be said that the tendency to them is some hundreds of times greater in the fourth year than at birth;" and that about twenty-seven in one hundred of those who die before puberty are affected with tuberculous disease. These are startling facts, and indicate error in some part or other of our management of the young, which it is the special duty of the medical profession to find out and provide against. In early life, when digestion and nutrition are most active, the abdominal organs and glands seem to be most frequently affected; whereas, after the age of eighteen, the tubercles most commonly affect the lungs. In both, however, the disease is the same, and its seat alone is different.

Among the trades and occupations in which mankind are engaged, those which tend directly to deteriorate the general health are invariably those which are most productive of tubercular disease; thus affording another proof of the latter being a constitutional and not a local ailment. "The effect of sedentary habits,"

says Dr. Clark, "in all classes and conditions of society, is, in my opinion, most pernicious; and there is perhaps no cause, not even excepting hereditary predisposition, which exerts such a decided influence in the production of consumption, as the privation of fresh air and exercise. Indeed, the result of my enquiries leads to the conviction that sedentary habits are among the most powerful causes of tuberculous disease, and that they operate in the higher classes as the principal cause of its greater frequency among females." (P. 201.) The truth of this opinion is confirmed by daily observation; and, in accordance with it, we find that shoemakers, tailors, dressmakers, weavers, and those who pass their days in a constrained position, in ill-ventilated apartments,—and whose occupations "are eminently calculated to prevent the free exercise of the respiratory organs, to diminish the powers of the circulation, to impair the nutritive functions, and produce a corresponding depression of the nervous energy," and deterioration of the general health,—are among the most frequent sufferers from consumption: whereas, butchers, tanners, seamen, and others, who live actively in the open air, and are well fed and clothed, are almost exempt, although they are often exposed to cold and wet, and to all those vicissitudes of the weather which are generally regarded as the immediate causes of phthisis. The Jesuits were well aware of the bad consequences of inactivity, and, with their usual sagacity, made it a rule of the order that there should be some active bodily exertion after every two hours of study.

The total insufficiency of even the most active local causes to produce tubercular phthisis, where *there is no predisposition,* is strikingly apparent in the fact of stonemasons, needle-pointers, dry-grinders, and other operatives, who are constantly exposed to the inhalation of acrid dust into the lungs, and who are actually carried off, most of them before the age of forty, by pulmonary disease so induced; but in whom, nevertheless, it is rare to meet with true consumption. Almost always it is chronic bronchitis, a distinctly inflammatory affection, which proves fatal to them; and tubercles appear only where a strong predisposition exists. Nothing can shew more clearly the paramount share which the constitutional state has in giving rise to the disease.

*A cold, moist, and variable climate* is extremely favorable to the production of scrofula and consumption; and evidently exerts its influence chiefly on the general system. In Lepelletier's division this cause is included among those which act chiefly by impeding excretion; and its agency is exemplified in the frequency of scrofulous and tubercular affections towards the end of winter and the beginning of spring, when the absence of the healthful stimulus of solar life and warmth has had time to operate.

Such, then, are the true causes of consumption; and the colds and inflammations, to which it is commonly ascribed, are merely the sparks which set fire to the train, and owe their power of doing mischief solely to the constitution being already prepared. The tuberculous predisposition is truly the first act in the fatal drama; and therefore, in guarding against and watching its approach, the greatest vigilance and decision are required, especially towards the approach of puberty. For, as has been well remarked by our author, that period of life "is one of great importance in persons predisposed to consumption."

"If the health has suffered by mismanagement in education, or from other causes, during early youth, the system very often shews it in a remarkable manner about the period of puberty. The development of the body, which should naturally take place at this age, and which in healthy persons is accompanied with an increase of strength and vigour in the system, is often delayed beyond the usual period, or imperfectly accomplished. If, therefore, young persons remain weak and thin, or look unhealthy after the usual period of puberty, they may be considered as in great danger of falling into tuberculous cachexia. Those who have been overworked at school, or kept much at sedentary occupations, frequently present this state of deteriorated health.

"Under such circumstances, the utmost care will be necessary to prevent tuberculous disease. A strict examination and enquiry should be made into the state of every function, and more especially of those connected with nutrition. The condition of the digestive organs and skin requires particular attention, because they are most commonly deranged; the tongue will very often be found furred; the alvine evacuations irregular; and the skin dry, harsh, and affected with eruptions, particularly with acne in its various forms; in young females the catamenia are retarded or imperfectly established. Such are the common symptoms presented to us in these cases, but they admit of considerable variety in different constitutions and temperaments.

"The absolute necessity of early attention to those indications of tuberculous cachexia cannot be too strongly impressed by medical men upon the minds of parents. There can be no doubt that a very large proportion of our youth, who fall victims to consumption from twenty to thirty years of age, might be saved by a timely adoption of the simple measures detailed in this chapter, and which are, in some degree, within the power and reach of all." (P. 296.)

It may be thought that we, as well as Dr. Clark, insist too much on the constitutional causes of phthisis; but, in a practical point of view, everything depends on a thorough acquaintance with them. If we are influenced by the prevailing opinion that phthisis is a merely local affection of the lungs, independent of any constitutional condition, we necessarily remain inactive till the tuberculous deposit has taken place, and the disease becomes avowedly incurable: whereas, if, from conviction, we adopt the principle that it is the pre-existing diathesis alone which gives power to the exciting causes, we are as necessarily led to take alarm at the earliest moment, and to use every means in our power, while a successful result is still possible.

Following the scope of Dr. Clark's enquiry, rather than his actual arrangement, and passing over in silence such parts of the subject as have been already fully discussed in the excellent works of Laennec, Andral, and Louis, we shall now shortly consider some of the consequences which flow from the pathological views so ably expounded by him.

If it be true that phthisis is the result of a pre-existing morbid state of the constitution, either inherited from the parents or produced by external circumstances, and that ordinary causes rarely suffice to produce it where the predisposition does not exist, it unavoidably follows that *the prevention and removal of that predisposition* must assume a preponderating importance in the eyes of every reflecting practitioner; and that, to the attainment of these objects, even more than to the cure of phthisis, the attention of the physician ought to be especially directed.

After the full exposition we have given of the causes of tuberculous cachexia, the principles of the preventive treatment may be despatched in a few words. The grand object is to secure observance of the laws of health so far as to avoid the errors which we have pointed out as inducing the tendency to the disease; whence the reason is obvious why a thorough investigation of the causes, and of their mode of operation, becomes so necessary in practice. If the predisposition arises from the bad health or imperfect constitution of the parents, the obvious dictate of nature and of the moral law is that parents so constituted ought not to marry, as they have no right to entail a curse on their offspring. If, again, the child acquire the predisposition from insufficiency or excess of food, confinement in a vitiated atmosphere, or living in a low, damp, and cold locality, let these causes be thoroughly remedied, and health will follow. In like manner, if excessive study, indulgence in sedentary habits, depression of mind, dyspeptic disorder, or other debilitating cause, be in operation, the most efficacious preventive treatment will consist in placing the person in a more favorable situation, and in adopting every measure calculated to improve the general health. Among such measures, free exhilarating exercise in the open air,—in games, on horseback and on foot,—adequate clothing,—nourishing diet,—living in a cheerful and airy situation, —the full play of the lungs in reading, recitation, and conversation, —and the avoidance of cold, wet, fatigue or exposure to night air,— will be especially serviceable. From long experience, we strongly recommend that the shoulders, top of the chest, and spine be kept comfortably warm; as nothing tends more to prevent congestion of the upper lobe of the lungs and irritating cough. The ordinary dress in Britain is in this respect very defective. In the house and in public places females have little or no covering on the neck and shoulders, although out of doors the warm shawls worn in winter are a great protection. The dress of males is also much open to objection. Generally speaking, the coat and waistcoat are open in front, and expose the chest to the cold; and even when a close double-breasted waistcoat is worn, the back of both it and the coat is left without lining, and presents only one fold; although there are perhaps three or four in front. The consequence is, that the spinal column, which ought to be completely protected, remains comparatively exposed, and the nerves issuing from it become impaired in vigour, and not only increase the delicacy of the chest, but lead to disordered digestion and induce sluggishness of the bowels; which in their turn react on the general health. We can at least affirm that we have experienced, and occasionally seen, this result; and that it has generally been removed or diminished in intensity by removing the cause above alluded to.

Among the preventives of tuberculous cachexia, we concur with Dr. Clark in laying much stress on the systematic practice of full and deep inspiration, and on the direct exercise of the lungs in reading aloud and indulging in noisy amusements in the open air. It is astonishing how much may be done in this way to develope and give strength to the chest, and we think the profession overlook too much the advantages which may be derived from some of these simple expedients, under proper regulation.

It is especially towards puberty, as already remarked, that preventive treatment becomes so important. If proper care be then taken to invigorate the constitution, a long continuance of good health may often be secured, even under considerable subsequent exposure. In general, the precautionary treatment is adopted too late, is not decided enough in its character, or is not systematically persevered in for a sufficient length of time. Instead of being encouraged to continued cure by an increase of strength and health, the patient and his friends often think they may now relax in their efforts, as the necessity has become less urgent than before. Having for several years past acted on this principle enforced by Dr. Clark, we could point to more than one instance in which a timely abandonment, for a year or two, of all scholastic and sedentary pursuits, for active exercise in the open air, regular living, and removal to the country in summer, have led to the permanent reestablishment of health in scrofulous fast-growing youths, who previously presented every appearance of soon becoming the victims of tubercular disease. It is in this stage of life that the rich have so much in their power, by removing for two or three years to a warmer and steadier climate, and adopting a rational system of physical and mental education, in place of the long school-hours and intellectual cramming and emulation now so fashionable, and so detrimental to health.

Most of the recommendations given by Dr. Clark for the prevention of phthisis are equally applicable to the treatment of tubercular cachexia; and one evil of the extent to which the author has subdivided his subjects is, that some of his less reflecting readers will fail to have this fact sufficiently impressed upon their minds. For example, the choice of a favorable locality, change of air, exercise on horseback, cheerful mental occupation, attention to clothing, and every thing, in short, which we have already spoken of as constituting a part of the *preventive* treatment, is equally important in the *cure* of tuberculous cachexia. Yet in the chapter dedicated to the latter subject, with the exception of two pages on climate, sailing, and travelling, there is no reference made to any of them as conditions which require to be fulfilled; and medical remedies only, such as alteratives, tonics, and purgatives, are noticed. The careful reader will not suffer from the omission, but when we consider that this is the only curable stage of consumption,—that Dr. Clark's treatise will necessarily become a book of reference and consultation both to the medical attendant and to the invalid,—and that, in *referring* to a work, we generally read at the time only the part in which we are more immediately interested, or in which information is wanted,—we cannot help regretting that the author did not embody in the beginning of the chapter a strong and decided statement of the necessity of attending to these indispensable conditions of recovery. Phthsis, in its second stage, is so confessedly intractable, that we should consider the treatment of the preceding cachexia as demanding more ample and careful development than that of the disease itself; and therefore, when the second edition shall be required, we trust that Dr. Clark will so far improve his arrangement as to collect every thing bearing upon the prevention and cure of the cachexia into one focus, and leave nothing to be sought for by the reader from the other chapters. It is better that a useful truth should be even thrice repeated, than that it should not be found in the place where it is most wanted. We are the more anxious on this point, because we are as deeply impressed as Dr. Clark himself with the conviction that in a large proportion of cases the disease *may* be prevented, although it cannot be cured; and we think his work otherwise excellently calculated not only to extend this conviction among the profession, but to assist in converting the possibility into a reality. The zeal and success, indeed, with which Dr. Clark has advocated the efficacy of preventive measures, and shewn their application to constitute a most important and hitherto neglected part of the province of the physician and of the duty of parents, are perfectly admirably. Years will no doubt elapse before society will reap the full benefit of the projected improvement; but every day is bringing us nearer the point when the family practitioner will be called upon to direct the physical and general education of the young, and to exercise a more efficient superintendence over public health, with a view to *prevent* as well as to *cure* disease. And were any additional evidence required to prove how much may be thus accomplished, the single circumstance of one class of society being much more free from consumption than another, would be sufficient to shew that we already possess the means of warding it off to a much greater extent than our own indolent ignorance is willing to admit, and that we are proportionnally culpable in neglecting to employ them.

We had marked for comment various passages descriptive of the features, pathology, and curative treatment of tuberculous disease, but have left no room for them. We regard, however, this omission as unimportant, compared with the duty of giving a correct view of the principles on which the work is written. If we have done justice to this part of the subject, few of our readers will fail to study the original treatise. The author's observations on the remedies used in the cure or alleviation of phthisis are characterized by soundness, discrimination, and judgment; but we can refer only to those on the use of emetics, which we think deserve serious attention. Dr. Clark mentions that emetics have been extensively employed, in every stage of the disease, in one of the military hospitals of Naples, by Dr. Giovanni de Vittis, and with astonishing success. If the cases treated by Giovanni de Vittis were really cases of phthisis, and if the results be correctly reported, it would be right for some of our enlightened hospital physicians to lose no time in giving his mode of treatment a fair trial, especially in the earlier stages of the disease. We confess that, looking to the origin and nature of phthisis, we cannot see cause to be sanguine in our expectations. At the same time, in a case like this, where every thing that has been hitherto tried has almost uniformly failed, we think that any plan which holds out even the slightest encouragement ought to be fairly tested, and its real powers widely proclaimed. In chronic bronchitis resembling consumption, emetics are unquestionably useful, and it *may be* that in phthisis also they are beneficial.

In drawing to a close this imperfect notice of one of the most valuable works which have lately appeared, we must do the author the justice to say that he lays no claim to originality in the views which he has advocated. "Much that is contained in the following pages," he modestly says, "is already known to the more intelligent and experienced of the profession; and the only credit I can claim is the having, perhaps, placed the subject in a more striking point of view, and advocated it with an earnestness commensurate with its importance." To this credit, and to more than this, Dr. Clark is most fully entitled. If he has not been the first to originate the doctrines which he expounds, he has unquestionably been the first to appreciate and make known their true value, and to demonstrate their easy applicability to the diminution and prevention of one of the most prevalent and fatal diseases to which mankind is subjected. And in public estimation, this latter service often, we suspect, ranks higher than the former. The mere discovery of a truth is of little use unless its importance also is perceived and made extensively known; and this has not hitherto been the case with the peculiar views which constitute the staple of Dr. Clark's publication. We may add that, to the general as well as to the professional reader, the work will prove of the deepest interest, and its perusal of unequivocal advantage.

